# Illegal performance enhancing drugs and doping in sport: a picture-based brief implicit association test for measuring athletes’ attitudes

**DOI:** 10.1186/1747-597X-9-7

**Published:** 2014-01-30

**Authors:** Ralf Brand, Philipp Heck, Matthias Ziegler

**Affiliations:** 1 Department of Sport and Exercise Psychology, University Potsdam, Am Neuen Palais 10, Potsdam 14469, Germany; 2 Psychological Institute, Humboldt University of Berlin, Unter den Linden 6, Berlin 10099, Germany

**Keywords:** Doping attitude, Bodybuilding, Indirect test, Implicit attitude test (IAT), Methodology

## Abstract

**Background:**

Doping attitude is a key variable in predicting athletes’ intention to use forbidden performance enhancing drugs. Indirect reaction-time based attitude tests, such as the implicit association test, conceal the ultimate goal of measurement from the participant better than questionnaires. Indirect tests are especially useful when socially sensitive constructs such as attitudes towards doping need to be described. The present study serves the development and validation of a novel picture-based brief implicit association test (BIAT) for testing athletes’ attitudes towards doping in sport. It shall provide the basis for a transnationally compatible research instrument able to harmonize anti-doping research efforts.

**Method:**

Following a known-group differences validation strategy, the doping attitudes of 43 athletes from bodybuilding (representative for a highly doping prone sport) and handball (as a contrast group) were compared using the picture-based doping-BIAT. The Performance Enhancement Attitude Scale (PEAS) was employed as a corresponding direct measure in order to additionally validate the results.

**Results:**

As expected, in the group of bodybuilders, indirectly measured doping attitudes as tested with the picture-based doping-BIAT were significantly less negative (η^2^ = .11). The doping-BIAT and PEAS scores correlated significantly at *r* = .50 for bodybuilders, and not significantly at *r* = .36 for handball players. There was a low error rate (7%) and a satisfactory internal consistency (*r*_*tt*_ = .66) for the picture-based doping-BIAT.

**Conclusions:**

The picture-based doping-BIAT constitutes a psychometrically tested method, ready to be adopted by the international research community. The test can be administered via the internet. All test material is available “open source”. The test might be implemented, for example, as a new effect-measure in the evaluation of prevention programs.

## Background

Doping in sport, defined as the presence of a prohibited substance or its metabolites or markers in an athlete’s sample, or evidence of the attempted use or use of a prohibited method [1], appears to be widespread [2]. For example, recent analyses of biochemical data from 2,737 elite track and field athletes revealed incidents of blood doping in an average of 14% of athletes, with up to 48% positive samples in athletes from particular countries [3]. It is thus astonishing that for example in the year 2011, only 1.2% of the 243,193 test samples, which have been analyzed in accredited WADA (World Anti-Doping Agency) laboratories, produced adverse analytical findings [4]. Humble detection rates like this might have contributed to what has been described as a shift from detection-based deterrence to prevention-based deterrence of doping in sport [5,6].

Evaluations of doping prevention programs raise the problem (among others) that open interviews with participating athletes, for example whether or how much their attitude towards doping has changed as a result of the prevention program, do not always lead to conclusive information. Especially, the tendency to respond in a socially desirable manner (the athlete knows what the interviewer expects to be the “right” answer) has a negative effect on the validity of respective self-report data [7]. For example, it has been shown that answers indicated in a standardized questionnaire, from athletes who exhibited positive doping results in biochemical hair analyses, allowed no conclusions about their factual doping behavior [8]. When it comes to detecting changes in the behavior of athletes who participated in a doping prevention program, it may be of limited value to solely rely on data from such direct inquiries therefore. However, by far the largest part of all existing studies on the effectiveness of doping prevention programs in sport is based on such self-reports [9].

### Direct and indirect testing of athletes’ doping attitudes

There is an increasing number of empirical studies, in which (sets of) cognitive determinants of the intention to dope, or more seldom the behavior itself, have been investigated [10-12]. Most of them include and several have been devoted to the investigation of doping attitudes. This is because most psychologists will concur that attitude is one of the most important cognitive predictors of intention and/or behavior [13].

Three fundamental characteristics can be used to essentially define the psychological construct of attitude [14]. First, attitudes fulfill an evaluative task in assessing objects and persons. Second, important attitudes are represented in a person’s memory. Third, attitudes can have affective, cognitive and behavioral causes, as well as bring about changes in affect, cognition and behavior themselves. Current social-cognitive theories of behavior (dual system models, as for example the Reflective Impulsive Model, RIM) assume that reflective as well as automatic cognitive processes influence behavior [15]. It is thus important to differentiate between explicit and implicit attitudinal components [16]. Explicit attitudes are evaluative judgments about an attitude object that result from processes of conscious, deliberate thought. They can be measured by using direct tests, for example standardized attitude questionnaires. In contrast, implicit attitudes are evaluative reactions resulting from spontaneous cognitive associations, which are automatically activated by a relevant stimulus [17]. Reaction-time based indirect tests have been shown to be the method of choice for measuring this attitudinal component.

The Performance Enhancement Attitude Scale, PEAS [18], is one of the most widely used direct tests to measure (thus explicit) doping attitudes [19,20]. This questionnaire contains items such as “doping is necessary to be competitive”, which has to be evaluated on a 6-point Likert-type scale ranging from “strongly disagree” to “strongly agree”.

Only very few studies have attempted to measure implicit attitudes related to doping in sport [8,21,22]. All of these drew on variations of one reaction time-based indirect test, the Implicit Association Test, IAT [23].

The IAT represents one class of tests, which are based on the theoretical assumption that knowledge is stored in our memory in a “networked” fashion. Nodes represent knowledge in an associative network of semantic information. If a node of the network is activated, this activation spreads within the network and associated nodes are co-activated automatically [24]. IATs take advantage of the fact that the activation of an attitude object (of a semantic network node; e.g. through presentation of the drug name “Erythropoietin/EPO”, which can be used as a doping substance) automatically activates the evaluation associated with this attitude object, e.g. “positive” or “negative”.

The IAT is typically presented as a lexical sorting task (speed test) on the computer where two concepts (one target and one evaluative) are mapped on the same response key of the computer’s keyboard. The sorting task is easier (and thus requires less time; a few hundred milliseconds on average) when the two concepts sharing the same response key (e.g. doping and dislike) are closely associated than when the two concepts on the same key are not associated (doping and like). Several different ways of calculating test scores have been proposed [25]. All of these approaches use the difference in response time between related and unrelated pairs, which is then interpreted as a measure of the associative strength between the target concept and attribute characteristics.

It is important to mention that there is controversy about the implicitness of the processes measured by the IAT and its test variants [26]. It is beyond doubt at the other hand that the test has evolved as one of the standard measures of implicit attitudes in social cognition research [27,28]. Recognizing the notwithstanding well-founded critique on the IAT, we will refer to it as an established *indirect test* throughout this paper, and avoid a language suggesting that IAT test scores would reflect unbiased (pure) implicit processes.

### Validating IATs

Researchers have argued in favor of reaction-time based indirect tests that, compared with questionnaires (direct tests), are easier to conceal the ultimate goal of measurement from the participant [29]. Therefore, the IAT may be less susceptible to the social desirability bias. On the other hand, critics have argued that one weakness of the IAT is that it is sometimes difficult to prove its validity. Non-associative factors, like for example cognitive skills, familiarity and perceptual similarity of stimuli, are able to bias the measurement and could also be used to explain observed differences in reaction-times therefore [30]. Arguments like these counter one fundamental assumption of the IAT, namely that differences in response-times correspond only to the associative strength between the categories. Another critique is that deliberate faking of the IAT seems to be possible, especially if participants are informed about the test’s general setup and content [31]. Aside from that, until today, results from numerous studies indicate that the IAT is a sensible methodological choice for attitude measurement when socially sensitive information is under question, e.g. gender stereotypes or prejudices against other social groups [28,32-35].

The method of choice for validating a doping IAT certainly consists of indirectly measuring the doping attitudes of athletes who have verifiably taken an illegal substance, and compare them with those of athletes who have verifiably not. The indirect test then should reveal more positive doping attitudes in the first group than in the second group. The results of one pilot study points in this direction. Petróczi et al. showed that athletes who were found guilty of taking doping substances on the basis of biochemical hair analyses indicated more positive attitudes in an IAT than athletes who tested negative [8]. However, these results are based on an extremely small sample (6 dopers vs. 4 non-dopers) and the method of biochemical testing may have been suboptimal. For example it is unclear from the article whether a quantitative or qualitative differentiation of endogenous hormones and externally introduced substances was carried out.

Insofar as the biochemical test for the use of doping substances, which should ideally be used as an external criterion for validating experimental results, is very costly and, especially, since it is not easy to find doping positive athletes who would participate in such studies, other researchers have employed variations of this known-group validation strategy.

Petróczi, Aidman, and Nepusz expected, for example, that athletes who are regularly involved in competitions would exhibit a stronger dislike for doping [22]. Rather unexpectedly, they could not find this difference. A study by Lotz and Hagemann, by contrast, suggested a group difference between more (bodybuilding and track-and-field) or less (handball and table tennis) doping prone sports [35]. However, in their study, the indirectly measured attitude scores correlated with a random factor.

There is evidence that IAT results are strongly dependent on the test stimuli used [36]. So one reason for the inconclusive evidence in these two initial studies on doping IATs [22,35] may be that they used suboptimal test stimuli. Results from one later methodological study, which found substantial error rates and adaptational learning effects associated with both IATs, point into this direction [21]. Another or an additional reason may be that the between-group variance of IAT scores was marginal in the first [22] as well as in the second study [35]. Both have found fairly negative evaluations of doping (doping attitudes) in all groups.

### The present research

This article introduces a picture-based brief-IAT (BIAT) for the indirect measurement of athletes’ doping attitudes. BIATs have already been used successfully in various other behavioral contexts [37,38]. This methodological (brief) variant of the standard IAT contains considerably fewer (less than half) sorting trials than the standard IAT procedure, resulting in much shorter testing time (less than five minutes). We expect the development of such a time efficient variant to bring about improved test compliance on the side of the participants.

The here presented doping-BIAT uses pictures instead of word stimuli. Considerations for the use of pictures instead of word stimuli go back to the early years of IAT research [23,39]. Picture (B)IATs appear to produce slightly smaller IAT effects than word (B)IATs [40]. One possible explanation lies in the different representation levels of the stimuli [41]. Words are more abstract and elicit more associations (personal images) than concrete pictures. Despite this potential drawback, we focus our development efforts on the use of pictures as stimuli. The main reason for this is that we expect this to facilitate the applicability and further examination of this doping-BIAT beyond language barriers.

The picture-based doping-BIAT will be validated using a known-group validation strategy [22,35]. A group of athletes is approached, for which the probability of finding a greater number of subjects with positive doping attitudes is particularly high: We address bodybuilders who regularly participate in bodybuilding competitions. Studies concerning the prevalence of doping in this sport suggest that up to 40% of these athletes regularly consume doping substances [42,43]. Using anabolic steroids for example, is seen as an integral part of this sport’s culture among most athletes [44]. More importantly, several researchers have illustrated that bodybuilders are comparably open to confess their abuse. The doping attitudes of bodybuilders are compared with those of handball players. Unfortunately, one cannot be sure that handball has a *de facto* low prevalence of doping, although this is suggested by the very low figures in recent WADA reports [4]. Due to the handball association’s strict official anti-doping policy, we expect to have a much better chance of sampling handball players with comparably less positive attitudes towards using doping substances anyhow. This choice, handball vs. bodybuilding, was supposed to maximize the between-group variance in the participating athletes’ (B)IAT scores.

We expect that both doping attitude tests, the direct as well as the indirect one, unsheathe significantly more positive personal evaluations of doping in the group of bodybuilders than in the group of handball players. This is considered an empirical indicator for the picture-based doping-BIAT being capable of providing valid information about athletes’ doping attitudes.

## Methods

### Picture-based doping-BIAT

The BIAT is a computer-based testing method for the indirect measurement of attitudes [37]. Stimuli from four categories are presented on a computer monitor and have to be sorted as quickly as possible to the right or left by pressing either “I” or “R” on the computer keyboard, depending on the task specific instructions. The reaction-times and correctness are measured.

This standard setup was adopted for our picture-based doping-BIAT [37]. It combines the concept classifications (four categories) of *doping* (focal) vs. *health food* (unfocal) with the attribute classifications *like* vs. *dislike*. In one of the doping-BIAT’s two combined task blocks, stimuli either representing the concept *doping* or the attribute *like* must be sorted to the right by pressing the “I” key (block A). In the other combined task (block B), *doping* stimuli and stimuli representing the attribute category *dislike* go together, and have to be sorted to the right by pressing the “I” key.

At the beginning of each of the two test blocks (combined task blocks A and B), the subjects are shown the complete stimulus set of the categories on two introductory screens (*doping* and *like* on one, *doping* and *dislike* on the next screen. The stimuli of the non-focal category, *health food* are not shown). During the test blocks, the task relevant category labels (*doping* and *like*, or *doping* and *dislike*) remain visible at the top and bottom of the screen. Test stimuli are presented between the two labels. Each test block contains 20 trials of which the first four are for practice. According to the notation of Sriram and Greenwald, our procedure corresponds to a doping–dislike/like–(health food) BIAT [37].

The order of the two test blocks was counterbalanced across participants. In the case of an incorrect response, a red X appeared on the screen and the participant had to correct his answer. Only the test trials of the two test blocks were included in the evaluation, i.e. per person there are reaction-times from two blocks with 16 trials each. All reported results refer to this total of 32 trials per person.

Average differences in reaction times and resulting difference values were calculated according to the D4 algorithm [25]. This includes that reaction times above 10,000 ms and those of error trials are deleted and are replaced by an error value (average reaction-time of this participant in all correct trials of the block plus 600 ms; mere elimination of error trials would have a negative impact on the reliability of the already short test). Subsequently, the average response times are calculated for both blocks and offset against each other ([*doping* and *dislike*] – [*doping* and *like*]). The resulting difference is divided by the pooled intraindividual standard deviation of all reaction times from both blocks. The calculated *D-score* is thus adjusted for intraindividual differences in motor responsiveness and varies between -2 and +2. D-scores below zero indicate that stimulus categorization took longer for the [*doping* and *like*] combination than for the [*doping* and *dislike*] combination. According to the logic of the test, an average group D-score below zero thus means that these subjects associate the concept of doping with *dislike* rather than with *like*, indicating a negative evaluation of doping attitude (i.e. doping attitude). The test was programmed using the Inquisit 3.0 software and performed on a conventional 17” laptop with QWERTZ keyboard. The two response buttons “I” and “R” were highlighted with stickers.

Testing of the psychometric properties of BIATs is still comparatively rare [25,45]. Test reliability scores vary greatly (internal consistencies: Cronbach’s α = .54 to .94; test-retest reliability: *r* = .17 to .71) between different BIATs [37].

All picture stimuli used in the proposed picture-based doping-BIAT are available from an online stock photography database.^a^ In a preliminary study, 32 raters were asked to evaluate the preselected images with regard to their associative strengths towards their reference constructs. The 16 images with the highest associative strengths were then adopted for the doping-BIAT. In its final version the picture-based doping BIAT uses four photos representing doping substances (pictures with various pills, ampoules, syringes; focal target concept), four photos with health food (apple, cereal, vegetables, salad; unfocal contrasting concept), four different emoticons with positive facial expressions (“smileys”; evaluative category), and four emoticons with negative expressions (disaffected or resentful; evaluative category).

### PEAS

The PEAS is a 17-item uni-dimensional questionnaire [18]. Sample items are “legalizing performance enhancements would be beneficial for sports”, “athletes are pressured to take performance-enhancing drugs”, or “only the quality of performance should matter, not the way athletes achieve it”. Statements are rated on a 6-point Likert scale from 1 = “strongly disagree” to 6 = “strongly agree” (neutral responses are not possible). PEAS scores may therefore range between 17 and 102 (with a theoretical mean of 59.5). Higher scores indicate more positive evaluations of doping (i.e. doping attitudes). The scale was presented as a paper and pencil version in our study.

There is detailed information on psychometric test properties [18]. The PEAS exhibited acceptable to good internal consistency (α = .71 - .91) and acceptable test-retest reliabilities (*r* > .75) in this review. Its convergent validity was examined using a known-group approach. Athletes who admitted to doping were expected to have higher total PEAS scores than honest, clean athletes. All studies, except one, showed that this was true. The effects of the group differences were partly very small (*d* = 0.11), partly large (*d* = 1.39), resulting in a moderate average effect (*d* = 0.71).

### Personal information questionnaire

Age, height and weight of all participants were collected to describe the study sample. The body mass index (BMI) is used to illustrate the physical appearance, particularly of the bodybuilder subsample. As recent research has indicated that (B)IAT-scores may be biased by participants’ cognitive skills a proxy for this variable was included [46]. Participants were asked how long they have attended school in adolescence (coded as 1 = basic schooling to 9^th^ grade, 2 = high school degree after 10^th^ grade, 3 = general qualification for university entrance).

### Sample

All participants (*N* = 43, including 21 bodybuilders and 22 handballers) were male. The average age was 31.0 years for the bodybuilders (*SD* = 10.2) and 25.4 years for the handball players (*SD* = 7.7). While three of the bodybuilders had left school after 9^th^ grade, nine after 10^th^ grade, and an additional nine after completing the general qualification for university entrance, only one handball player had left school after 9^th^ grade, three after 10^th^ grade, and 18 after completing the general qualification for university entrance.

All handball players reported to participate in competitions. The same was true for all bodybuilders. An additional criterion for the latter group was that they had been training at least four days a week for at least one year. This criterion was established to ensure that only those bodybuilders are chosen for testing who had already concerned themselves intensively with the issue of muscle building and who had thus most likely dealt with doping issues. On average, the bodybuilders had been working-out regularly for 12.9 years (*SD* = 10.4), the handball players for 14.0 years (*SD* = 6.1). The average BMI of the bodybuilders was 32.7 (*SD* = 3.8), that of the handball players was 24.7 (*SD* = 2.5). The BMI values of the bodybuilders illustrate their immense muscle mass (i.e. contrary to what the BMI scale would normally suggest, in the case of the bodybuilders, these results do not point to adiposity).

### Procedure

The bodybuilders were recruited in two bodybuilding studios and tested directly on site. The handball players were recruited during the training sessions of a handball club and also tested on site. Both samples were thus convenience samples. The picture-based doping-BIAT was always completed before the personal information questionnaire, which was followed by the PEAS. During the assessment, a study assistant was present, so that any questions could be answered immediately. Prior to participating, the athletes were guaranteed anonymity. They were informed about the procedure and purpose of the study (i.e. comparing bodybuilders’ and handball players’ attitudes towards doping), and their formal informed consent was obtained. Ethical approval for the study was granted by the University of Potsdam.

### Statistical analysis

All variables were subjected to simple descriptive statistics first (calculation of mean scores and standard deviations, score distribution analysis). Chi-square (education) and t-statistics (age) were performed to check for group differences between handball players and bodybuilders. Then, an analysis of the BIAT error trials was carried out, and reliability coefficients for both measuring instruments (PEAS and doping-BIAT) were calculated. Bivariate partial correlations between direct (PEAS) and indirect attitude tests were formed. Two separate analyses of covariance (ANCOVA) were planned as main statistical analyses, using either the PEAS or the BIAT score as dependent variables, group as the factor variable, and age and education as covariates. With the exception of these ANCOVAs (in which two-tailed tests were used), one-tailed tests for statistical significance tests were carried out (with an alpha level of .05). Effect sizes were interpreted according to Cohen’s conventions. SPSS 19.0 and 20.0 were used for all statistical analyses.

## Results

### Subsample characteristics

The two subsamples differ statistically significant in terms of education, χ^2^ = 6.78, *p* = .03 (Fisher’s exact test), and age, *t*(41) = 2.04, *p* = .05, *d* = 1.03. Both are representing meaningful g-factor variables able to bias IAT results [46,47], and have to be statistically addressed (by analyses of covariance) in all further data analyses.

### Description of measurements from the indirect attitude test (doping BIAT)

In our picture-based doping BIAT, there were no trials with reaction times below 300 ms or above 1,000 ms. They were thus within the expected limits and there were no critical values that had to be replaced or deleted. The analysis of incorrect trials (7%) revealed that participants made an average of 2.1 mistakes (max. = 10; min. = 0; *SD* = 2.2). Of these, 23% were made in the first test block and 77% in the second test block. This is expectable because the subjects in the second test block have to reverse the just learned categorization of stimuli. 46% of mistakes are made in the focal category, 54% in the non-focal category. The bodybuilders made more mistakes than the handball players (60% vs. 40%). Mistakes occurred more frequently (66%) in block A [doping + like] than in block B (34%). This illustrates a general tendency that it was more difficult for our participants to combine the concepts of *doping* and *like*.

As recommended by Sriram and Greenwald [37], the reliability of the BIAT was calculated using the split-half method. A correction using the Spearman-Brown formula resulted in a reliability of *corr r*_*tt*_ = .66 for our picture-based doping-BIAT.

**Figure 1:**
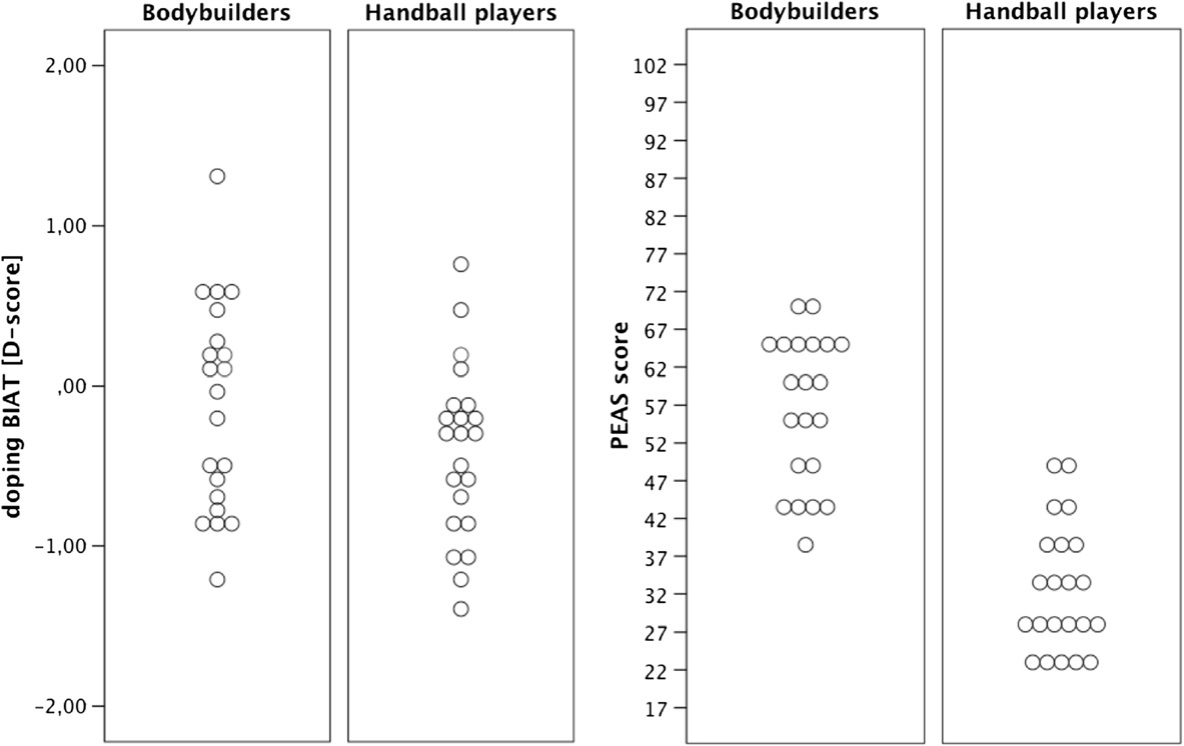
Distributions of individual BIAT (left part; indirect attitude test) and PEAS (right part; direct attitude test) doping attitude scores.

**Figure 2:**
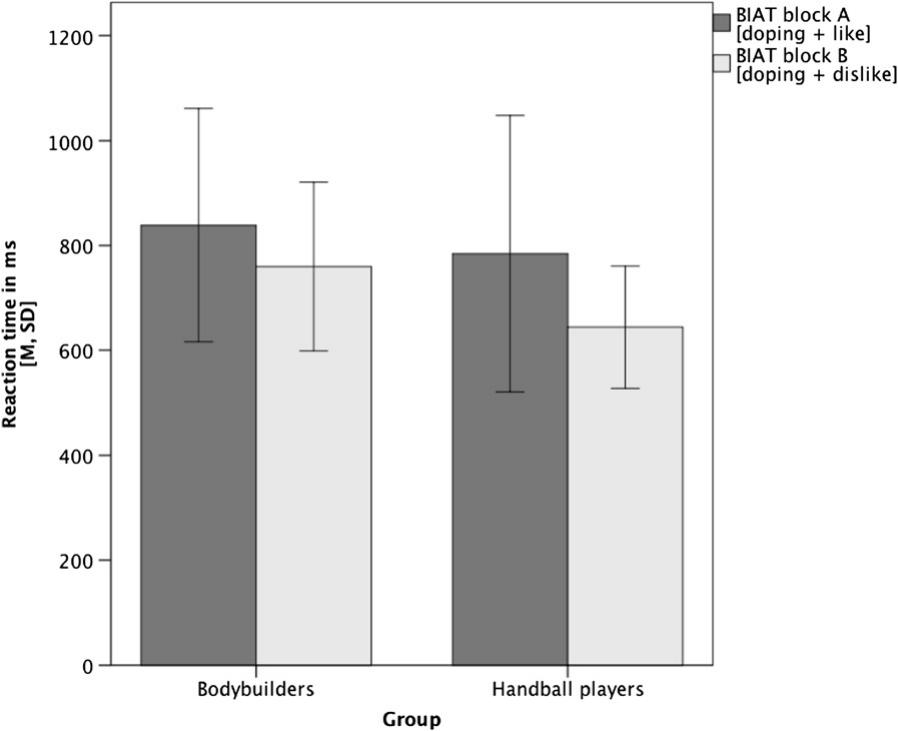
Illustration of the BIAT-effect (comparison of within-group differences in mean reaction time latencies [block B minus block A]).

Detailed descriptive information on all reaction-time measurements is given in Table 1. In addition, distributions of individual scores within groups (Figure 1, left part) and average reaction time differences between blocks and groups (BIAT-effect; Figure 2) are graphically illustrated.

**Table 1:** Results from the direct (PEAS questionnaire) and indirect (picture-based doping BIAT) tests of the bodybuilders’ and handball players’ doping attitudes

	**Bodybuilders [*n*** **= 21]**	**Handball players [*n*** **= 22]**
PEAS questionnaire [mean score]	56.2 (± 8.7)	32.7 (± 8.7)
Picture-based doping BIAT		
D-score [mean score]	-0.14 (± 0.65)	-0.40 (± 0.54)
Block A [mean RT]	838 ms (± 222)	748 ms (± 263)
Block B [mean RT]	759 ms (± 161)	644 ms (± 116)
All blocks [mean RT]	799 ms (± 189)	714 ms (± 189)

Averaged across all blocks, the handball players exhibited shorter (79 ms on average) response time differences between blocks than the bodybuilders (140 ms). Both groups of athletes were faster in block B [*doping* and *dislike*] than in block A [*doping* and *like*].

These differences between blocks were used to calculate the D-values. On the group mean level these indicate a dislike of doping (negative D-values) in handball players as well as in bodybuilders. Nevertheless 14 participants (of 43) had a positive D-value. These subjects were faster in block A [*doping* and *good*] and thereby showed a relative preference for doping. Of these 14 participants, 10 were bodybuilders and 4 were handball players. The mean between-group difference in D-scores was 0.26, with the handball players having a lower average group score than the bodybuilders. This points towards a less negative evaluation of doping (doping attitude) in the group of bodybuilders.

### Description of measurements from the direct attitude test (PEAS)

A Cronbach’s-α of .85 indicates good internal consistency of the PEAS scale in our sample. Only items 11 and 12, both of them relate to recreational drugs, show comparatively low item-scale correlations (*r* = .15 and *r* = -.06, respectively). Similar problems with these items are described in previous studies [48]. However, as the whole scale’s internal consistency in our sample remains acceptable, and to facilitate comparisons with results from other studies most of all, these two items were maintained for the PEAS score calculation.

Table 1 encloses relevant descriptive information on the two groups’ PEAS mean scores. The distribution of individual mean scores is depicted in the right part of Figure 1.

The average attitude score towards doping of all participants (as measured with the PEAS) is 44.2. The bodybuilders achieve an average score of 56.2, which is very close to the theoretical mean of the scale. Their responses can thus be semantically anchored between the item response labels “slightly disagree” and “slightly agree”. The handball player’s average score is considerably lower (-23.56), indicating that they “strongly disagree” with almost all PEAS statements.

### Correlations between attitude tests

The partial correlation (controlled for age and education) between directly (PEAS) and indirectly tested (picture-based doping BIAT) attitude scores was considerably greater for the bodybuilders (*r* = .50, *p* = .02) than for the handball players (*r* = .36, *p* = .07).

### ANCOVAs

Levene’s test for equality of error variance showed that the group variances neither differed for the PEAS nor for the doping-BIAT D-scores, *F*_*PEAS*_(1,41) = .73, *p* = .40 and *F*_*D*_(1,41) = 1.82, *p* = .18, respectively.

Group membership exerted a significant and moderate to large effect on the doping-BIAT D-score, *F*(1,38) = 4.8, *p* = .04, η^2^ = .11; 1-β = .57. The estimated mean group score difference (after having controlled for the two covariates age and education) was *D* = 0.42, *p* = .02, *d* = 0.71. On the group mean level handball player’s doping attitude was thus more negative (rejection of doping) than that of bodybuilders.

There was a significant and very large effect for the factor group on the direct doping attitude test’s results (PEAS score), *F*(1,38) = 58.9, *p* < .01, η^2^ = .61; 1-β = 1.0. Estimated mean group scores (after having controlled for the two covariates age and education) differed significantly by 24.6 points, *p* = < .01, *d* = 2.6. Again, handball players exhibited a more negative evaluation of doping (doping attitude) than bodybuilders.

## Discussion

Compared with handball players, bodybuilders’ exhibited less negative evaluations of doping in the reaction-time based indirect as well as in the direct attitude test. The doping-BIAT (indirect test) and PEAS (direct test) scores are moderately correlated. The reliability and error rate analyses of the BIAT scores indicate that with regard to stimuli selection the general setup of this test seems to be adequate. In sum, these results point to the validity of the proposed picture-based BIAT for measuring athletes’ doping attitudes. This indirect test might therefore serve as an alternative or additional instrument to questionnaire-based direct tests, which could be more open to be distorted by social desirability biases and faking attempts.

These results are discussed and conclusions shall be drawn with regard to two specific issues. As this article’s emphasis is on test and psychometric properties of the picture-based doping BIAT, this issue will be discussed in detail first. Then, as this study’s history of ideas is rooted in social science anti-doping research, we think it is worthwhile embedding our results (i.e., the potential of the proposed doping BIAT as a testing instrument) into current discussions of the role of attitudes and intention in doping behavior.

### Test and psychometric properties

Bodybuilders evaluated doping significantly less negatively in the BIAT (*D* = -0.14) than handball players (*D* = -0.40). This group difference is reflected even more clearly in the direct attitude test where the bodybuilders reach a mean PEAS score of 56.2 and the handball players one of 32.7. PEAS- and D-scores correlated positively in both groups with a medium effect size. This correlation is more pronounced in the group of bodybuilders (*r* = .50, *p* = .02) than in the group of handball players (*r* = .34, *p* = .07). This is particularly interesting, as in previous studies direct and indirect test measures have correlated only slightly. One meta-analysis from outside the doping area, in which results of 126 studies with indirect–direct test correlations were aggregated, reports a mean correlative strength of ρ = .24 between such measures [34]. According to the authors of this meta-analysis, correlations increase with the degree of conceptual similarity of the respective measures. The correlation decreases if this similarity is missing or when other important factors exert influence (e.g. lack of motivation, or lack of introspective ability). Most of all, the correlation is usually also lower when the tests ask about sensitive content (socially undesirable or illegal behavior. Our results correspond well with these meta-analytic findings. They should be considered as an indication of the (convergent) validity of our indirect test.

The observed direct–indirect correlation in our study becomes even more interesting when it is put in relation to one recently published finding [8]. In this study a (rather small) group of doping substance users (verified by biochemical tests) could be statistically characterized by a clear dissociation between direct test results (distinctively negative evaluation of doping) and indirect test results (rather positive doping attitude). The authors’ explanation for this dissociation was that athletes would either consciously decide against revealing their true attitude in the direct test or that they have no insight into their own “true” evaluation of doping (for example, because the automatic bias for social desirability makes accessing it more difficult). Especially in our group of bodybuilders there was no such dissociation. Looking at the group of handball players it is well possible that nobody in this group (according to our hypothesis) actually uses doping substances or methods. It is rather unlikely that the bodybuilder group contained no (or only few) users however. We therefore assume that the dissociation pattern in factual dopers reported by Petroczi et al. [8] is a domain-specific (sport disciplines) and setting-specific (e.g. trustworthiness of assurances of anonymity) phenomenon. The bodybuilders participating in our study did not run the risk of being criticized for their attitudes. Even if the athletes had admitted to use forbidden performance enhancing substances, they would have not been subject to any consequences because bodybuilding associations have not committed themselves to the WADA anti-doping policies (with the result that substance abuse is not prosecuted as an unsportsmanlike practice in this sport). The distorting influence of social desirability in this setting could have been rather low therefore. Future studies should provide information on the question in which settings and/or sports dissociation patterns should be expected or not (and, in which both types of attitude test therefore should be carried out in order to characterize users).

As well as the standard IAT procedure, the BIAT represents a relational measure, in which the attitude towards a target category (here: doping) is compared with a contrasting (in the BIAT, unfocal) category. Our BIAT results thus imply that bodybuilders as well as handball players had more positive attitudes towards health food than towards doping (this is similar to previous doping-IAT studies, in which the contrasting categories tea blends [21,35] or nutritional supplements [21,22] were used). This also means that for example the bodybuilders’ D-scores, wich are close to zero, might indicate that doping and food were rated as nearly equally positive (like) or negative (dislike). It would be very interesting to examine bodybuilders’ attitude towards health food with direct measures in follow-up studies therefore. This would serve to rule out the at least theoretically possible alternative explanation that in the proposed picture-based doping-BIAT the target and contrast categories’ valences were too similar (for bodybuilders at least).

Another methodological aspect is an important contribution of our study. All previous studies on the indirect doping attitude tests (IAT studies) used words as stimuli. Our main concern was to propose a doping-BIAT that could be used beyond international barriers by using pictures (photos) as stimuli that are understandable independent of one’s native language.

The error analysis in the BIAT shows that the use of images (as well as the new reference category, health food) had no noticeable influence on the participants’ test performance. The rate of 93% correctly sorted trials corresponds to findings from other BIAT studies [41], and suggests that the categories and stimuli could be easily distinguished. Moreover, it can be assumed that the error rate could have been minimized further if the study had been carried out in the laboratory (all measurements were carried out in the field, at the athletes’ training facilities). It can be concluded that in this doping-BIAT, assigning images instead of words, was a successful idea. With regard to our data, the benefits of images as stimuli (intuitive understanding, possibility of application across language barriers) are thus hardly accompanied by disadvantages (smaller effect sizes). Since the presented BIAT is mainly aimed at its possible use as an evaluation method for doping prevention programs, this international applicability represents significant added value. It is the first published non-verbal test method for measuring doping attitudes.

The diagnostic quality of a (B)IAT is also reflected in the test’s reliability. IATs typically have lower internal consistencies (between α = .65 and .75) than questionnaires [49]. As test reliability is mathematically affected by the number of test items (trials), it is not surprising that brief IATs exhibit even lower internal consistencies as standard IATs. Recently reported values in studies with significantly longer doping-IATs have been of similar values, with *r*_*tt*_ = .66 [22] or between α = .53 and .79 [21]. The reliability of our picture-based doping-BIAT (*r*_*tt*_ = .66) is thus within the acceptable range and constitutes a satisfactory result.

### The role of attitude and intention in doping behavior

Most models would not directly link attitudes with behavior. For example in the Theory of Planned Behavior (TPB) the both are linked through the mechanism of intention [50]. Not very surprisingly doping attitudes are thus able to predict doping intentions in elite athletes [10]. Additional variables such as reference group influences, threat appraisal, benefit appraisal, personal morality and legitimacy, or personality have been shown to predict athletes’ doping attitudes as well as their intentions [51]. There is one longitudinal study with nonprofessional and adolescent athletes showing that the TPB variables attitude, subjective norms and perceived behavioral control predict doping intentions as well as behavior [52]. But it is important to recognize that meta-analyses from other behavioral domains (e.g. health behavior) indicate that not more than 25-30% [53] of the behavioral variance can be explained by social-cognitive variables from this line of modeling.

Another, more recent, theoretical approach is that of dual system models. The Reflective-Impulsive Model is one proponent of this approach [15]. This model proposes that a reflective system of social information processing weighs knowledge for example about the athlete’s personal values or probabilities of behavioral consequences. As a result of this relatively slow process a behavioral option is chosen before an intention for the (planned) behavior is formed. The impulsive system, on the other hand, allows for parallel (automatic) processing in associative semantic networks. Cognitive associations that arise quickly and involuntarily after just the exposition to (e.g.) an attitude object, activate further elements of the network. Behavioral schemata can be directly initiated via this non-reflective route. Another approach to address the parallel processing of information in two interconnected systems arose with the Meta-Cognitive Model of Attitudes [54,55]. This proposes three different patterns of an implicit-explicit attitudes interplay (additive, interactive and double dissociation), and provides a framework to better understand the ways in which both forms of attitudes contribute to decisions in their own unique but inter-related way.

Implicit association tests, including the here presented picture-based doping-BIAT, are thought to reflect such non-reflective, impulsive processes. Without playing down the legitimate critique that (B)IATs might be restricted in terms of their capability to exactly mirror these processes [26], there is ample reason to assume that the (B)IAT represents an acceptable means to tap into the individual’s automatic evaluative reactions [56].

There is at least preliminary evidence that doping behavior also relies on implicit-explicit interplays, namely that it is associated with a possible dissociation of reflective and impulsive processes [8]. Doping prevention and intervention efforts, so far, tend to assume that doping behavior is a rather deliberate choice between competing alternatives. Introducing psychometrically tested methods such as the doping-BIAT, is prerequisite for taking a closer look at what happens in the impulsive system. This, in turn, will have implications on anti-doping policies or intervention [57].

### Limitations

Our known-group approach for validating the picture-based doping-BIAT is different from the approach, which was used to validate the PEAS [18]. The respective PEAS study compared the doping attitudes of declared users vs. non-users. Our approach is founded “only” in the hypothesis that bodybuilders are more tolerant with regard to doping issues than handball players. It is a necessary next step to investigate the predictive value of the picture-based doping-BIAT, for factual doping use. As open self-reports about use/nonuse of doping substances are likely to be biased by social desirability, e.g. biochemical doping tests should be used as criteria variables.

Alternatives to the (B)IAT, which by some authors are considered to be more promising, should be considered in the future. One criticism concerning the (B)IAT’s two test blocks is that recoding processes may have considerable impact on its results. This means that, when the stimuli are assigned to categories in the blocks, occurring differences in reaction-times might be influenced by different recoding speeds, and not only by the attitudes regarding the respective category. Rothermund and Wentura [45] therefore perceive as advantageous IAT variants that forego the two-block structure, for example the IAT-RF (recoding free) or the SB-IAT (single-block). Very recently, a sophisticated statistical algorithm has been proposed, which could help to disentangle the disparate contributions of associations and recoding in standard IAT procedures [58]. Integrating this algorithm in available (B)IAT analysis scripts is complex for the average user so far. Undoubtedly though, the continuous advancement of IAT methodology should be recognized by the anti-doping research community and rigorously incorporated whenever the tradeoffs between methodological complexity and possible earnings for test users in the applied field are in good balance.

The picture stimuli of the present doping-BIAT were pretested (i.e. associative strengths with the implied constructs were evaluated) in a German sample. In principle, although unlikely, it is possible that the selected pictures trigger different associations in participants from other nationalities or cultures [36]. This alternative explanation should be suspended through comparative intercultural studies.

Finally, the test should be applied (maybe as a side measure) in future studies, to analyze whether factors such as sample size, outlier values from some study participants in the direct as well as in the indirect attitude test, or the low reliability of some PEAS items have influenced the power of our statistical analyses.

## Conclusions

When designing our test, we tried to create conditions that would allow the picture-based doping-BIAT to quickly be adopted, examined and, most of all, applied outside of our work group. The stimulus material of this test is freely available on the Internet to anyone who wants to use it. The use of special software (Inquisit™, in our case) allows for online test presentation, giving work groups that do not have special software for rendering reaction-time based tests a chance to use it in cooperation networks (for example with our team). Therefore, we hope that the present study helps to create conditions for establishing an international standard of reference that promotes and renders more likely the use of indirect testing methods, for example, as a means of determining the effects of doping prevention measures on athletes’ attitudes.

## Endnote

^a^All stimuli from http://www.bigstockphoto.com. Registry numbers are 5641690, 15498974, 592197, 5101044 (doping); 167404, 1569998, 1455972, 14352824 (healthfood); 2870882, 7528186, 14462246, 14462315 (emoticons, smileys); 7904622, 259438, 3036237, 7060500 (emoticons, “dislike”).

## Competing interests

The authors declare that they have no competing interests.

## Authors’ contributions

RB, PH and MZ designed the study. PH and MZ conducted the statistical calculations, RB wrote the first draft of the manuscript. All three authors then jointly worked on all subsequent versions of the manuscript. All three authors read and approved the final manuscript.
